# Pregnant and breastfeeding women: A priority population for HIV viral load monitoring

**DOI:** 10.1371/journal.pmed.1002375

**Published:** 2017-08-15

**Authors:** Landon Myer, Shaffiq Essajee, Laura N. Broyles, D. Heather Watts, Maia Lesosky, Wafaa M. El-Sadr, Elaine J. Abrams

**Affiliations:** 1 Division of Epidemiology & Biostatistics, School of Public Health & Family Medicine, University of Cape Town, Cape Town, South Africa; 2 Centre for Infectious Diseases Epidemiology & Research, School of Public Health & Family Medicine, University of Cape Town, Cape Town, South Africa; 3 HIV Department, World Health Organization, Geneva, Switzerland; 4 Division of Global HIV and TB, Center for Global Health, Centers for Disease Control and Prevention (CDC), Atlanta, Georgia, United States of America; 5 Office of the Global AIDS Coordinator and Health Diplomacy, US Department of State, Washington DC, United States of America; 6 ICAP at Columbia University, Mailman School of Public Health, New York, New York, United States of America; 7 College of Physicians & Surgeons, Columbia University, New York, New York, United States of America

## Abstract

Landon Myer and colleagues discuss viral load monitoring for pregnant HIV-positive women and those breastfeeding; ART treatments can suppress viral load and are key to preventing transmission to the child.

With more than 18 million HIV-infected individuals having initiated antiretroviral therapy (ART) in low- and middle-income countries (LMICs) by the end of 2016, ensuring effective HIV care and treatment services is a global public health priority [1]. Viral load (VL) quantification provides a direct measure of the effectiveness of ART, with a consistently elevated VL suggesting poor adherence or treatment failure and the need for intervention. In turn, HIV VL monitoring is now recognised as a key component of ART services in LMICs in World Health Organization (WHO) guidelines, with an emphasis on scaling up access to VL testing for ART programmes [2].

Pregnant and postpartum women are an important population within ART programmes. In many countries, the majority of identified HIV-infected adults are women, and many women of reproductive age are diagnosed with HIV infection during pregnancy through prevention of mother-to-child transmission of HIV (PMTCT) services in antenatal care (ANC) [3]. With universal eligibility for ART for all HIV-infected pregnant and postpartum women (based on the WHO’s 2013 ‘Option B+’ policy [4]), many women of reproductive age initiating ART do so during pregnancy. PMTCT services extend through early infant diagnosis around 6–10 weeks postpartum until the cessation of breastfeeding and documentation of the infant’s final HIV testing status, which may extend well beyond 1 year postpartum based on the recently updated infant feeding recommendations [5]. With ongoing risk of HIV transmission throughout breastfeeding, maintaining ART adherence and viral suppression is especially crucial during this period.

Although the importance of routine VL monitoring for HIV-infected individuals on ART is widely recognised [6], there has been minimal attention to VL monitoring in pregnancy and the postpartum period. Here we discuss key considerations for VL monitoring in pregnant and breastfeeding women in the context of expanding access to VL monitoring (summarised in Box 1).

**Box 1. Summary of key issues in viral load monitoring for pregnant and breastfeeding women**

**Rationale for a priority population:**
- Reduce mother-to-child transmission (MTCT) risk
- Reduce maternal morbidity and mortality
- Reduce partner transmission risk
- Sustain long-term health of children and families

**Recognising distinct groups of pregnant women receiving antiretroviral therapy (ART):**
- Women already on ART at time of conception and entry into prevention of MTCT (PMTCT) services
- Women newly initiating ART in pregnancy

**Optimal timing of viral load (VL) testing during pregnancy and breastfeeding:**
- Dependent upon duration of ART use, laboratory turnaround times, and available interventions
- Modified intervals and more frequent testing during pregnancy and breastfeeding

**Urgency of responding to elevated VL during pregnancy and breastfeeding:**
- Time-limited period to reduce transmission risk from conception through delivery and breast feeding
- A high proportion of transmissions occur in late pregnancy, during delivery, and in the early postpartum period
- Objective to achieve viral suppression before delivery and maintain it during breastfeeding
- Pregnancy and particularly postpartum period as high risk for inadequate adherence

**Interventions for elevated VL during pregnancy and breastfeeding:**
- Routine World Health Organisation algorithms for management of elevated VL may be inadequate
- Protease inhibitor-based second-line treatment may be poorly tolerated during pregnancy
- Opportunity to enhance infant prophylaxis with multidrug antiretroviral (ARV) regimen if maternal VL not suppressed
- VL testing 4 weeks before delivery provides a basic approach to help target interventions from birth

## Importance of monitoring VL in pregnant and breastfeeding women

VL monitoring to ensure viral suppression in pregnant and breastfeeding women carries unique potential benefits for maternal, child, and family health. With viral suppression, ART reduces morbidity and mortality in HIV-infected individuals [7] and decreases the risk of secondary transmission [8]. The level of maternal viraemia is directly proportional to the risk of vertical transmission, and that risk is substantially increased within a narrow VL range, even with ART use [9]. Monitoring VL in pregnant and breastfeeding women is therefore crucial to keeping infants HIV free, maintaining maternal health, and promoting the health and well-being of families [10].

Despite the importance of ART for VL suppression during pregnancy and breastfeeding, there is mounting evidence that ART adherence may be inadequate during these critical periods [11,12]. Consequently, VL suppression may be suboptimal, leading to higher risk of MTCT and maternal disease progression [13,14]. The drivers of nonadherence are variable and may include ART side effects or pill burden in the context of pregnancy; increased psychosocial stressors related to pregnancy or caregiving; fragmented health systems that may require women to attend different clinics or transfer services after delivery; and inadequate patient counselling on the importance of VL suppression, particularly during breastfeeding [15]. In the context of broader efforts to help improve women’s retention in ART services during pregnancy and postpartum, VL monitoring to reinforce ART adherence in this population may have substantial benefits [16].

## Recognising distinct groups of pregnant women receiving ART

There are 2 distinct populations of HIV-infected pregnant and postpartum women that warrant separate attention for VL monitoring. Traditional PMTCT services are well equipped to provide HIV testing and counselling, initiate women on ART, and follow them up during pregnancy—including women who acquire HIV infection during pregnancy or postpartum. However, with increases in HIV testing and ART coverage at a population level, a growing proportion of HIV-infected women of reproductive age becoming pregnant have initiated ART before conceiving and thus enter ANC already on ART [17]. These women do not fit in the traditional scope of PMTCT services and warrant particular consideration. Ideally, women on ART will have received VL monitoring and have suppressed VL before conception, but this is not always the case [18]. And although effective use of ART before conception and during the period of MTCT risk is associated with an extremely low risk of vertical transmission, not all women on ART will remain suppressed throughout pregnancy and breastfeeding [13], necessitating ongoing monitoring.

## Optimal timing of VL testing in pregnancy and breastfeeding

Determining when VL should be measured during pregnancy and breastfeeding requires recognition of the specific population of women on ART who are being tested. For pregnant women entering ANC already on ART, VL testing to verify suppression should be considered early in pregnancy. For women initiating ART during pregnancy, women may be presumed to be viraemic until an appropriate time on ART has elapsed to allow for viral suppression, usually 2–3 months. For both groups, VL monitoring during pregnancy can help guide providers to institute more intensive adherence interventions and, if necessary, further clinical action with the goal of achieving viral suppression during labour and delivery. Ongoing monitoring throughout breastfeeding is warranted to help minimize postpartum transmission risks; half of all MTCT occurs during breastfeeding, making this is a critical time for ongoing viral suppression [19]. Importantly, the application of standard VL guidelines for nonpregnant adults, which recommend VL testing only 6–12 monthly, to populations of pregnant and breastfeeding women would pass over these critical periods for a large proportion of women, presenting important missed opportunities for preventing paediatric HIV infections.

The timing of VL monitoring will also be influenced by the effective turnaround time from specimen collection to result return, as well as the intervention strategies prescribed for patients with an elevated VL. In settings with long turnaround times, VL testing earlier in pregnancy may be required if interventions at delivery are intended, while more rapid turnaround times may enable monitoring to take place later during pregnancy. Point-of-care VL testing—which could effectively eliminate turnaround times—may play an important role in this context, but operational experience is needed. More generally, understanding the optimal timing and frequency of VL monitoring in pregnant and breastfeeding women requires urgent investigation; current WHO guidelines suggest that a VL conducted 4 weeks prior to delivery may be used to target enhanced infant prophylaxis, and this provides one approach that may be feasible in many settings.

## The urgency of responding to elevated VL during pregnancy and breastfeeding

Pregnancy and breastfeeding are time-limited periods, and several analyses have demonstrated increased transmission risks with each additional week of uncontrolled viraemia during pregnancy [20], underscoring the importance of timely testing, the rapid return of test results, and prompt clinical intervention in response to an elevated VL. Unfortunately, substantial delays may be introduced across the multiple steps of the VL monitoring ‘cascade’, and there is a need to minimize the time for each step, from initial specimen collection to testing, return of results, and appropriate clinical action. In many LMICs, delays in the VL ‘cascade’ may be on the order of weeks to months, a timeframe that is especially unacceptable in pregnancy and breastfeeding. The need to expedite each step of the VL cascade to identify and manage viraemia during high-risk transmission periods presents an important hurdle to realising the full benefits of VL monitoring in this population. From a health systems perspective, there are important opportunities to build on the platform and innovations introduced to scale up early infant diagnostic testing—such as tools to expedite the return of test results to facilities and patients—to accelerate the introduction of VL for pregnant and postpartum women [21].

## Interventions for elevated VL in pregnancy and breastfeeding

In children and nonpregnant adults, the WHO’s clinical algorithm for responding to an elevated VL test result, typically defined as >1,000 copies/mL, calls for provision of enhanced adherence counselling and repeat VL testing approximately 3 months after establishing adherence [2]. If the second test also shows an elevated VL, treatment failure is assumed, and a second-line ART regimen is initiated with the goal of achieving viral suppression. Though this approach is suitable for nonpregnant populations, it requires adaptation in the case of pregnant and breastfeeding women for 2 reasons.

First, the timeframe used for this clinical algorithm is not appropriate in the context of pregnancy and breastfeeding given the ongoing risk of MTCT. Ideally, an initial raised VL in a pregnant or breastfeeding woman should be detected and followed up within days with adherence support, and VL testing repeated within weeks to confirm VL reductions. In addition, the standard approach to the management of treatment failure, comprised primarily of ongoing adherence support and a change to second-line ART regimens, should be approached with caution in pregnancy and switching minimized if possible. In many countries, the recommended protease inhibitor (PI)-based second-line ART regimen has an increased pill burden and greater side effect profile than first-line regimens; it is possible that the underlying challenges of treatment adherence that drive apparent treatment failure in many settings may be exacerbated with the use of these regimens. And though newer classes of antiretroviral drugs such as integrase inhibitors have favourable side effect profiles and rapid antiviral activity that make them particularly appealing during pregnancy, clinical experience, safety data in pregnancy, and regulatory approvals are required.

Second, an elevated VL in a pregnant or breastfeeding woman should result in additional interventions being considered for women and their infants, apart from a switch in the ART regimen. As outlined in recent WHO guidelines, an elevated VL late in pregnancy may be used to target enhanced antiretroviral prophylaxis regimens for HIV-exposed infants [2]. Similarly, targeted HIV testing at birth for ‘high risk’ infants may be directed by maternal VL during gestation. Of note, some interventions (such as targeted infant prophylaxis) may be appropriate after a single elevated VL test result, but others may require repeated VL testing over time (such as changing to a second-line ART regimen).

While the optimal intervention package in response to an elevated VL during pregnancy or breastfeeding will depend on the local health systems context, the clear delineation of intervention strategies is fundamental to the design of a VL monitoring programme. Ultimately, if reliable and timely VL results are not available, or no clinical intervention is undertaken in response to an elevated VL result, there is limited utility to conducting VL monitoring.

## Emerging research questions

Given the range of issues that require consideration in VL monitoring for pregnant and breastfeeding women, there are major programmatic questions that require attention. These questions give rise to an emerging research agenda that cuts across clinical, behavioural, and programmatic domains, underpinned by the need to understand the aetiology, detection, and optimal management of viraemia in this population (Table 1). In addressing these questions, simulation studies and modelling may play an important role in helping to understand the possible costs and benefits of different scenarios for both maternal and child health. Ultimately, understanding the impact of VL monitoring programmes at scale remains an important goal for HIV prevention and treatment programmes, and throughout, pregnant and breastfeeding women on ART are a priority population requiring specific consideration.

**Table 1:** Key clinical, behavioural, and health systems research questions related to viral load monitoring in pregnant and breastfeeding women.

Domain	Policy and programme issues	Specific research questions
Clinical	When to monitor viral load (VL) in pregnant and breastfeeding women?	When and how frequently should VL monitoring be conducted during pregnancy and breastfeeding to maximise the detection of elevated VL, balancing clinical benefits with costs and operational complexity?
	When and how to intervene against elevated VL?	What are the implications of low-level viraemia for transmission risk and maternal outcomes, and how will that impact the threshold for virologic failure in pregnant and breastfeeding women? What are the impacts of intervening against detectable VL < 1,000 copies/mL on maternal and infant outcomes?
		What are the relative contributions of antiretroviral resistance versus antiretroviral therapy (ART) nonadherence as immediate causes of elevated VL in pregnant and breastfeeding women?
		How does the epidemiology of HIV drug resistance in a given population modify the choice of interventions in response to elevated VL on routine monitoring in pregnancy?
		How can new antiretroviral agents alter the timing and frequency of VL monitoring, as well as responses to elevated VL, in pregnant and breastfeeding women?
		How can VL monitoring support adherence counselling for patients, including in response to elevated VL detected during routine monitoring?
Behavioural	What causes elevated VL in pregnant and breastfeeding women?	What are the drivers of ART nonadherence during pregnancy and breastfeeding?
		How does knowledge or understanding of VL as a concept, and an individual’s VL result at a particular timepoint, influence adherence behaviours and retention in care?
		Does routine VL monitoring contribute to improved adherence and suppression outcomes during pregnancy and breastfeeding?
Health systems	How can VL monitoring be implemented to support care for pregnant and breastfeeding women?	How can steps in the VL cascade be expedited to minimize delays from specimen collection to clinical action?
		What role may point-of-care VL monitoring technologies play in improving the detection and management of elevated VL?
		How can VL test results be optimally communicated across different health services providing care throughout pregnancy and breastfeeding?
		How do VL monitoring and feedback systems integrate into differentiated models of care?
		What role can VL monitoring and feedback systems play in monitoring and promoting long-term retention of postpartum women?

## Conclusion

Over the past 2 decades, global PMTCT efforts have emphasized women’s access and adherence to antiretroviral drugs as a major element of effective PMTCT programmes. With increasing scientific insights and programmatic sophistication, we are shifting to view viral suppression as a target of global policies, in PMTCT specifically and ART programmes more broadly. Reaping the long-term benefits of ART use in pregnant and breastfeeding women requires sustained viral suppression, and VL monitoring in pregnant and breastfeeding women has a critical role to play in ensuring effective ART use to reduce HIV transmission risk and achieve optimal maternal, child, and family health outcomes.

## Abbreviations

ANC: antenatal care
ART: antiretroviral therapy
ARV: antiretroviral
CDC: Centers for Disease Control and Prevention
LMICs: low- and middle-income countries
MTCT: mother-to-child transmission
PI: protease inhibitor
PMTCT: prevention of mother-to-child transmission of HIV
VL: viral load
WHO: World Health Organization
